# Serum mannan-binding lectin-associated serine proteases in early pregnancy for gestational diabetes in Chinese pregnant women

**DOI:** 10.3389/fendo.2023.1230244

**Published:** 2023-10-24

**Authors:** Ming Gao, Jing Li, Rui Zhang, Ninghua Li, Weiqin Li, Shuang Zhang, Peng Wang, Hui Wang, Zhongze Fang, Zhijie Yu, Gang Hu, Junhong Leng, Xilin Yang

**Affiliations:** ^1^ Department of Epidemiology and Biostatistics, School of Public Health, Tianjin Medical University, Tianjin, China; ^2^ Tianjin Key Laboratory of Environment, Nutrition and Public Health, Tianjin Medical University School of Public Health, Tianjin, China; ^3^ Tianjin Center for International Collaborative Research on Environment, Nutrition and Public Health, Tianjin Medical University School of Public Health, Tianjin, China; ^4^ Project Office, Tianjin Women and Children’s Health Center, Tianjin, China; ^5^ Department of Toxicology and Sanitary Chemistry, School of Public Health, Tianjin Medical University, Tianjin, China; ^6^ Population Cancer Research Program and Department of Pediatrics, Dalhousie University, Halifax, NS, Canada; ^7^ Chronic Disease Epidemiology Laboratory, Pennington Biomedical Research Center, Baton Rouge, LA, United States

**Keywords:** mannan-binding lectin-associated serine proteases, deoxycholic acid, glycoursodeoxycholic acid, lysophosphatidylcholines, gestational diabetes mellitus

## Abstract

**Aims:**

This study aimed to explore associations of mannan-binding lectin-associated serine protease (MASP) levels in early pregnancy with gestational diabetes mellitus (GDM). We also examined interactions of MASPs and deoxycholic acid (DCA)/glycoursodeoxycholic acid (GUDCA) for the GDM risk and whether the interactive effects if any on the GDM risk were mediated via lysophosphatidylcholine (LPC) 18:0.

**Materials and methods:**

A 1:1 case-control study (n = 414) nested in a prospective cohort of pregnant women was conducted in Tianjin, China. Binary conditional logistic regressions were performed to examine associations of MASPs with the GDM risk. Additive interaction measures were used to examine interactions between MASPs and DCA/GUDCA for the GDM risk. Mediation analyses and Sobel tests were used to examine mediation effects of LPC18:0 between the copresence of MASPs and DCA/GUDCA on the GDM risk.

**Results:**

High MASP-2 was independently associated with GDM [odds ratio (OR): 2.62, 95% confidence interval (CI): 1.44–4.77], while the effect of high MASP-1 on GDM was attributable to high MASP-2 (*P* for Sobel test: 0.003). Low DCA markedly increased the OR of high MASP-2 alone from 2.53 (1.10–5.85) up to 10.6 (4.22–26.4), with a significant additive interaction. In addition, high LPC18:0 played a significant mediating role in the links from low DCA to GDM and from the copresence of high MASP-2 and low DCA to GDM (*P* for Sobel test <0.001) but not in the link from high MASP-2 to GDM.

**Conclusions:**

High MASP-1 and MASP-2 in early pregnancy were associated with GDM in Chinese pregnant women. MASP-2 amplifies the risk of low DCA for GDM, which is mediated via LPC18:0.

## 1 Introduction

Gestational diabetes mellitus (GDM), a pregnancy-specific glucose intolerance, is one of the most prevalent metabolic disorders in pregnancy (1). According to the latest estimates of the International Diabetes Federation, approximately 14% of pregnant women worldwide were affected by GDM (2). Although GDM is a transient illness during pregnancy, it increases the risk of short-term and long-term complications for both mothers and their offspring (3). Our meta-analysis found that lifestyle interventions initiated before the 15th gestational week can only reduce the GDM risk by 20%, and lifestyle interventions initiated after the 15th gestational week are ineffective in reducing the risk (4). Hence, it is critically crucial to explore potential biomarkers for GDM predictions in early pregnancy in order to accurately identify pregnant women at high risk of GDM for effective interventions.

Mannan-binding lectin (MBL)-associated serine proteases (MASPs) are important components in the activation of the lectin pathway in the human complement system (5). When MBL recognizes and binds to mannose residues on the pathogen surface, activated MASPs can subsequently initiate the complement cascade through cleavage of complement proteins C2 and C4 to generate C3 and C5 convertases (6, 7). Indeed, overactivation of the complement system produces excessive inflammatory responses, resulting in β-cell function impairment and insulin resistance and thus glucose dyshomeostasis via activating the production of inflammatory mediators and stimulating macrophage infiltration (8, 9). In recent years, several studies have reported that increased MASPs were associated with an elevated risk of prediabetes and type 2 diabetes (10, 11). Given that GDM and type 2 diabetes share many common risk factors, it is likely that MASPs also play a role in the etiology of GDM, although the association has not been researched.

Our previous studies have reported that abnormal metabolites such as bile acids (BAs) and lysophosphatidylcholines (LPCs) were associated with an increased risk of GDM (12, 13). Interestingly, adjustment for high LPC18:0 slightly attenuated odds ratios (ORs) of low deoxycholic acid (DCA) and glycoursodeoxycholic acid (GUDCA) for GDM (12). BAs regulate lipid metabolism, ameliorate hepatic inflammation, and modulate metabolic homeostasis by regulating insulin secretion in pancreatic β cells (14). Indeed, MASPs can activate endothelial cells and trigger pro-inflammatory signaling and may be linked to chronic inflammatory diseases (15, 16). Given that insulin resistance is a consequence of low-grade chronic inflammatory state, the persistent inflammation caused by increased MASPs may be associated with reduced insulin sensitivity in pregnant women with GDM. It is worthwhile to examine the interrelationships among MASPs, BAs, and LPCs for better understanding the etiology of GDM. Specifically, BAs and MASPs may have a synergistic effect on the risk of GDM, possibly being mediated by downstream LPCs.

In the current study, we used a nested case-control design within a prospective cohort of pregnant women in Tianjin, China, aimed to explore: 1) the associations of MASP levels in early pregnancy with the risk of GDM; 2) the additive interactive effects between high MASPs and low DCA/GUDCA for the occurrence of GDM; and 3) whether LPC18:0 mediated interactive effects between high MASPs and low DCA/GUDCA if any on the risk of GDM.

## 2 Materials and methods

### 2.1 Study design and participants

The study design has been described previously (17). Briefly, 22,302 pregnant women from six central urban districts in Tianjin, China, were recruited at the first antenatal care visit and followed up to the delivery and postpartum period through the GDM screening and management system between October 2010 and August 2012. The study protocol was approved by the Ethics Committee for Clinical Research of Tianjin Women and Children’s Health Centre (TWCHC), and written informed consent was obtained from participants before data collection.

All pregnant women at the 24th–28th gestational weeks were offered a 50-g 1-h glucose challenge test (GCT) in non-fasting status at a primary care hospital. Pregnant women with a GCT value ≥7.8 mmol/L were referred to a central GDM clinic located within the TWCHC for a 75-g 2-h oral glucose tolerance test (OGTT) after at least 8 h of fasting. GDM was diagnosed using the International Association of Diabetes and Pregnancy Study Group (IADPSG)’s criteria (18).

Among 22,302 enrolled pregnant women, 2,991 women provided overnight fasting venous blood samples at their first antenatal care visit. Of them, 227 pregnant women were excluded due to lack of GCT results or lack of OGTT results if their GCT was ≥7.8 mmol/L. Among the remaining pregnant women, 243 women developed GDM and selected as the cases and 243 women without GDM matched on age ( ± 1 year) were selected as the controls. These women had received their first antenatal care at a median of 10.0 [interquartile range (IQR): 9.0–11.0] weeks of gestation. Among the 243 pairs of pregnant women, 207 pairs have complete metabolomics and DNA data. Of the remaining 36 pairs, 25 pairs of pregnant women were randomly selected for screening of proteins differentially expressed between the cases and the controls using a data-independent acquisition (DIA) assay. The differentially expressed proteins identified were published previously (19). The 207 pairs of pregnant women were used to validate the differences in the differentially expressed individual proteins between the 25 GDM cases and the 25 controls using a parallel reaction monitoring (PRM) assay.

### 2.2 Data collection procedures

Methods of detailed data collection have been described previously (17). Briefly, demographic and anthropometric data were collected via a series of structured questionnaires or retrieved from the medical records at the first antenatal care visit, the time of GCT until postpartum. Body weight measured at the first antenatal care visit was regarded as pre-pregnancy body weight because weight gain during the first trimester of pregnancy is slight (20). Body mass index (BMI) was calculated as body weight in kilograms divided by the square of height in meters. Weight gain to GCT was computed as the difference in body weight between the first antenatal care visit and the time of GCT.

### 2.3 Measurement of serum proteins

#### 2.3.1 Sample pretreatment for the PRM assay

Separated serum was stored at -80°C and thawed at 4°C when used. Quantitatively weigh 2 μL serum, add 195 μL lysis solution and shake until fully dissolved, then take the supernatant and reserve 10 μL for quantification. Samples were reduced with 5 mM DTT for 1 h at 37°C and subsequently alkylated with 10 mM iodoacetamide for 45 min at room temperature. The protein was digested with Trypsin Gold (Promega) at 1:50 enzyme-to-substrate ratio for 16 h at 37°C. The mixture sample (mix-sample) and the remaining peptides (single-sample) were all desalted with C18 cartridge to remove the high urea and dried by vacuum centrifugation.

#### 2.3.2 High-performance liquid chromatography fractionation

The mix-sample was fractionated using a C18 column (Waters BEH) on a Rigol L3000 high-performance liquid chromatography (HPLC) operating at 0.7 mL/min, with the column oven being set to 50°C. Gradient elution was developed using phase A [100% water, 0.1% formic acid (FA)] and phase B (100% acetonitrile, 0.1% FA). The eluates were monitored at UV 214 nm, collected for a tube per minute, and finally merged into three fractions. All fractions were dried under vacuum, then reconstituted in 0.1% (v/v) FA in water.

#### 2.3.3 Liquid chromatography-tandem mass spectrometry analysis

Samples were randomized prior to the mass spectrometry (MS) procedures. For transition library construction, shotgun proteomics analyses were performed using a Q Exactive HF-X mass spectrometer (Thermo Fisher) operating in the data-dependent acquisition (DDA) mode. A sample volume containing 1 μg of total peptides from the fraction sample reconstituted in 0.1% FA was injected onto a home-made C18 Nano-Trap column (2 cm × 100 μm, 3 μm). Peptides were separated on an analytical column (25 cm × 75 μm, 100 Å) using an 80-min linear gradient from 0% to 100% of eluent B at a flow rate of 600 nL/min. There was a single full-scan mass spectrum in the Orbitrap (350–1,500 m/z, 120,000 resolution) followed by data-dependent MS/MS scans in an ion-routing multipole at 27% normalized collision energy (NCE). The single-sample was reconstituted in 0.1% FA and injected onto U3000 UHPLC system (Thermo Fisher) coupled with a Q Exactive HF-X mass spectrometer (Thermo Fisher) operating in the PRM mode. The liquid conditions were the same as above. Parameters were set as follows: MS1 and MS2 resolution 60,000, scan range 150–2,000 m/z, and an NCE of 28%.

#### 2.3.4 Data processing

We carried out a correlation analysis to ensure the reliability of biological replicates. The samples showed excellent reproducibility with median Pearson correlation scores of biological replicates within case and control groups of 96.6% and 96.2%, respectively. The resulting MS data were analyzed by Skyline 21.1 software, and peak lists were searched against a UniProt Swiss-Prot Homo sapiens database (downloaded on 17 March 2022). Variable modifications: Oxidation (M), Acetyl (protein N-terminal); static modifications: Carbamidomethylation (C).

### 2.4 Measurement of serum BAs and LPCs

LC-MS/MS was used to identify and quantify the components of BAs and LPCs. The detailed measurement methods of BAs and LPCs have been described previously (12, 13).

### 2.5 Statistical analysis

All statistical analyses were performed using Statistical Analysis System (SAS), release 9.4 (SAS Institute Inc., Cary, NC, USA). A two-tailed *P* < 0.05 was considered to be statistically significant. The Shapiro–Wilk test was used to test normality for continuous variables. Continuous variables were presented as mean ± standard deviation (SD) or median (IQR). Differences between groups were compared using paired Student’s t-test or Wilcoxon signed-rank test. Categorical variables were presented as number (percentage) and compared using McNemar test or Fisher’s exact test.

Natural logarithm-transformed values were used to reduce the effect of skewness in the distribution of MASP levels. Restricted cubic spline (RCS) analyses with three knots at 0.05, 0.50, and 0.95 were performed to examine the linearity of associations of Ln MASP-1, Ln MASP-2 with the GDM risk. Because Ln MASP-1 was roughly linearly and Ln MASP-2 was roughly nonlinearly associated with the GDM risk, we stratified MASP-1 and MASP-2 into high vs. low levels, respectively, at its median and where the GDM risk started to increase steeply. This method including selection of cutoff points was described and used in many of our previous investigations (12, 19, 21). First, we performed conditional binary logistic regression to obtain the unadjusted ORs and 95% confidence interval (CI) of high vs. low MASP-1 and MASP-2 for the risk of GDM. Second, we adjusted for traditional GDM risk factors including pre-pregnancy BMI, family history of diabetes in first-degree relatives, systolic blood pressure (SBP), smoke before or during pregnancy, drink before or during pregnancy, weight gain to the time of GCT, preexisting morbidity (including heart diseases, nephritis, hepatitis, hyperthyroidism, anemia, and tumor), and multiple pregnancies. Finally, we adjusted for high MASP-2 in the MASP-1 model and high MASP-1 in the MASP-2 model. We further conducted mediation analysis to examine whether MASP-2 mediated the association between MASP-1 and GDM. Sobel tests were used to assess the statistical significance of the mediation effect (22).

In our previous analyses, we detected that DCA ≤0.28 nmol/mL, GUDCA ≤0.07 nmol/mL, and LPC18:0 ≥18.0 nmol/mL were independently associated with a markedly increased risk of GDM (12, 13). In this study, we used the same cutoff points to define low DCA, low GUDCA, and high LPC18:0. We tested the additive interaction between high MASP-2 and low DCA/GUDCA for GDM. Three measures, i.e., relative excess risk due to interaction (RERI), attributable proportion due to interaction (AP), and synergy index (SI), were used to judge the additive interactions. Any of RERI >0, AP >0, or SI >1 indicates a statistically significant additive interaction (23). Then, we conducted mediation analysis to examine whether high LPC18:0 mediated the association between the copresence of both high MASP-2 and low DCA, and GDM. Finally, receiver operating characteristic (ROC) curve analysis was used to assess the predictive values of serum MASP-1 and MASP-2 for GDM.

## 3 Results

### 3.1 Characteristics of the study participants

The mean age and BMI of the women at the first antenatal care visit were 29.2 [standard deviation (SD): 3.05] years and 23.0 (SD: 3.69) kg/m^2^. Compared with women without GDM, pregnant women with GDM had higher weight, BMI, SBP, and diastolic blood pressure (DBP) at the first antenatal care visit, higher gestational weeks and glucose level at the time of the GCT, and more likely to have a family history of diabetes in first-degree relatives. In the 25 pairs of GDM cases and controls, MASP-1 and MASP-2 were significantly higher in the GDM cases than those in the controls (means ± SD of Ln MASP-1 and MASP-2: 15.1 ± 0.26 vs. 15.0 ± 0.22, *P* = 0.029 and 15.8 ± 0.21 vs. 15.7 ± 0.19, *P* = 0.016, respectively). In the 207 pairs of GDM cases and controls, the levels of Ln MASP-1 and Ln MASP-2 were also higher in women with GDM than in those women without GDM (means ± SD of Ln MASP-1 and MASP-2: 14.1 ± 0.54 vs. 14.0 ± 0.60, *P* = 0.009 and 13.2 ± 0.65 vs. 13.0 ± 0.62, *P* < 0.001, respectively) (**Table 1**).

**Table 1:** Clinical characteristics of GDM and non-GDM women.

Characteristic	Non-GDM (n = 207)	GDM (n = 207)	*P* value
Variables at registration			
Age, years	29.2 ± 3.34	29.3 ± 2.74	0.477^*^
Height, cm	163.0 ± 4.54	163.3 ± 5.04	0.509^*^
Weight, kg	58.6 ± 9.78	63.9 ± 10.5	<0.001^*^
BMI, kg/m^2^	22.0 ± 3.46	24.0 ± 3.66	<0.001^*^
Systolic blood pressure, mmHg	104.2 ± 10.6	108.2 ± 10.5	<0.001^*^
Diastolic blood pressure, mmHg	67.9 ± 7.66	70.7 ± 7.93	<0.001^*^
Han ethnicity	200 (96.6)	202 (97.6)	0.564^†^
Education >12 years	113 (54.6)	109 (52.7)	0.683^†^
Parity ≥1	10 (4.83)	13 (6.28)	0.532^†^
Family history of diabetes in first-degree relatives	13 (6.28)	26 (12.6)	0.033^†^
Current smoker before or during pregnancy	13 (6.28)	15 (7.25)	0.695^†^
Alcohol drinker before or during pregnancy	52 (25.1)	64 (30.9)	0.190^†^
Serum proteins			
Ln MASP-1, relative units	14.0 ± 0.60	14.1 ± 0.54	0.009^*^
Ln MASP-1 ≥14.1 relative units	83 (40.1)	120 (58.0)	<0.001^†^
Ln MASP-2, relative units	13.0 ± 0.62	13.2 ± 0.65	<0.001^*^
Ln MASP-2 ≥13.4 relative units	43 (20.8)	91 (44.0)	<0.001^†^
Serum metabolites			
Bile acid metabolites			
DCA ≤0.28 nmol/mL	111 (53.6)	139 (67.2)	0.006^†^
GUDCA ≤0.07 nmol/mL	167 (80.7)	199 (96.1)	<0.001^†^
Lipids metabolites			
LPC18:0 ≥18.0 nmol/mL	40 (19.3)	175 (84.5)	<0.001^†^
Variables during pregnancy			
Gestational weeks at GCT, week	25.2 ± 2.28	25.0 ± 1.47	0.043^*^
Weight gain to GCT, kg/week	0.58 ± 0.21	0.57 ± 0.23	0.476^*^
GCT glucose, mmol/L	6.39 ± 1.35	9.30 ± 1.45	<0.001^*^

GDM, gestational diabetes mellitus; BMI, body mass index; MASP, mannan-binding lectin-associated serine protease; GUDCA, glycoursodeoxycholic acid; DCA, deoxycholic acid; LPC, lysophosphatidylcholine; GCT, glucose challenge test.

Data are reported in mean ± SD, median (interquartile range), or number (percentages).

^*^Derived from paired t-test or Wilcoxon signed-rank test.

^†^Derived from McNemar test or Fisher’s exact test.

### 3.2 Associations of Ln MASP-1 and Ln MASP-2 with GDM

**Figure 1:**
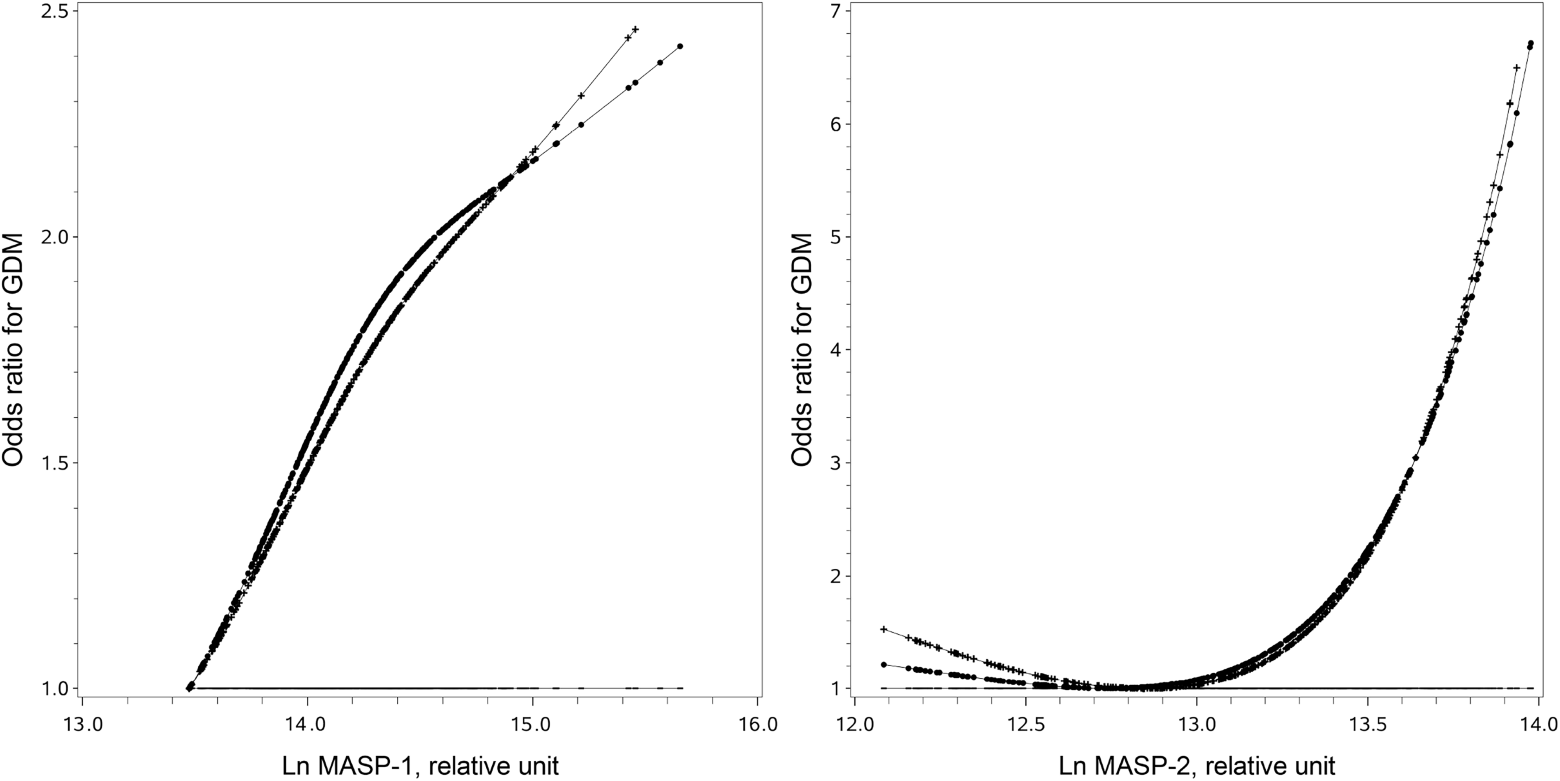
Full-range associations of individual mannan-binding lectin-associated serine protease 1 (MASP-1) and mannan-binding lectin-associated serine protease 2 (MASP-2) with the risk of gestational diabetes. The straight lines are the reference lines at odds ratio = 1. The dotted lines were derived from the univariate analysis. The crossed lines were derived from the multivariate analysis with adjustment for traditional risk factors, including pre-pregnancy body mass index, family history of diabetes in first-degree relatives, systolic blood pressure, smoke before or during pregnancy, drink before or during pregnancy, weight gain to the time of glucose challenge test, preexisting diseases (including heart diseases, nephritis, hepatitis, hyperthyroidism, anemia, and tumor), and multiple pregnancies.

Ln MASP-1 was positively associated with GDM in a linear manner (**Figure 1**). A high MASP-1 (i.e., ≥14.1 relative units) was associated with an increased risk of GDM in univariate analysis and multivariate analysis (OR: 1.97, 95% CI: 1.34–2.92 and 2.03, 1.31–3.16, respectively). Ln MASP-2 was positively associated with GDM in a nonlinear manner with a clear threshold effect (**Figure 1**). A high MASP-2 (i.e., ≥13.4 relative units) was associated with an increased risk of GDM in univariate analysis and multivariate analysis (OR: 3.29, 95% CI: 2.02–5.36 and 3.12, 1.82–5.35, respectively). After further adjustment for each other in multivariate analysis, high MASP-2 was independently associated with an increased risk of GDM, whereas the OR of high vs. low MASP-1 for GDM was no longer significant (OR: 2.62, 95% CI: 1.44-4.77 and 1.37, 0.83-2.29, respectively) (**Table 2**). The mediation analysis showed that MASP-2 mediated the association between MASP-1 and GDM (*P* for Sobel test: 0.003) (**Supplementary Table S1**).

**Table 2:** Odds ratios of MASP-1, MASP-2 for the risk of GDM.

	OR (95% CI)	*P* value
Univariate analysis		
Ln MASP-1 ≥ vs. <14.1, relative units	1.97 (1.34-2.92)	<0.001
Ln MASP-2 ≥ vs. <13.4, relative units	3.29 (2.02-5.36)	<0.001
Multivariate analysis 1		
Ln MASP-1 ≥ vs. <14.1, relative units	2.03 (1.31-3.16)	0.002
Ln MASP-2 ≥ vs. <13.4, relative units	3.12 (1.82-5.35)	<0.001
Multivariate analysis 2		
Ln MASP-1 ≥ vs. <14.1, relative units	1.37 (0.83-2.29)	0.221
Ln MASP-2 ≥ vs. <13.4, relative units	2.62 (1.44-4.77)	0.002

GDM, gestational diabetes mellitus; MASP, mannan-binding lectin-associated serine protease; OR, odds ratio; CI, confidence interval.Ln MASP-1 at 14.1 was chosen as its median while Ln MASP-2 at 13.4 was selected based on its odds ratio curve for GDM.Multivariate analysis 1 was adjusted for traditional risk factors, including pre-pregnancy body mass index, family history of diabetes in first-degree relatives, systolic blood pressure, smoke before or during pregnancy, drink before or during pregnancy, weight gain to the time of glucose challenge test, preexisting diseases (including heart diseases, nephritis, hepatitis, hyperthyroidism, anemia, and tumor), and multiple pregnancies.Multivariate analysis 2 was further adjusted for Ln MASP-2 ≥13.4 relative units in the MASP-1 model and Ln MASP-1 ≥14.1 relative units in the MASP-2 model.

### 3.3 Additive interaction between high MASP-2 and low DCA/GUDCA for GDM

Low MASP-2 (i.e., <13.4 relative units) and high DCA (i.e., >0.28 nmol/mL) used as the reference, the copresence of high MASP-2 and low DCA (i.e., ≤0.28 nmol/mL) enhanced the ORs of high MASP-2 alone and low DCA alone, respectively, from 2.53 (95% CI: 1.10–5.85) and 2.24 (1.21–4.17) up to 10.6 (4.22–26.4) for GDM. Additive interaction measures were significant (AP: 0.64, 95% CI: 0.33–0.95; and SI: 3.44, 95% CI: 1.19–9.94). Low MASP-2 and high GUDCA (i.e., >0.07 nmol/mL) used as the reference, the copresence of high MASP-2 and low GUDCA (i.e., ≤0.07 nmol/mL) enhanced the ORs of high MASP-2 alone and low GUDCA alone, respectively, from 12.2 (1.59–93.1) and 11.9 (2.33–60.4) up to 37.6 (6.92–204.6) for GDM. However, all of the three additive interaction measures were not significant (**Table 3**).

**Table 3:** Additive interactions between MASP-2 and DCA/GUDCA for the risk of GDM.

	Multivariate analysis 1		Multivariate analysis 2	
	OR (95% CI)	*P* value	OR (95% CI)	*P* value
Additive interaction between MASP-2 and DCA				
Ln MASP-2 <13.4 relative units and DCA >0.28 nmol/mL	Reference		Reference	
Ln MASP-2 <13.4 relative units and DCA ≤0.28 nmol/mL	1.66 (0.97-2.84)	0.063	2.24 (1.21-4.17)	0.011
Ln MASP-2 ≥13.4 relative units and DCA >0.28 nmol/mL	2.59 (1.21-5.53)	0.014	2.53 (1.10-5.85)	0.030
Ln MASP-2 ≥13.4 relative units and DCA ≤0.28 nmol/mL	8.45 (3.74-19.1)	<0.001	10.6 (4.22-26.4)	<0.001
RERI	5.20 (-0.91 to 11.3)	0.095	6.77 (-1.83 to 15.4)	0.123
AP	0.62 (0.30 to 0.93)	<0.001	0.64 (0.33 to 0.95)	<0.001
SI	3.31 (1.16 to 9.50)	0.026	3.44 (1.19 to 9.94)	0.023
Additive interaction between MASP-2 and GUDCA				
Ln MASP-2 <13.4 relative units and GUDCA >0.07 nmol/mL	Reference		Reference	
Ln MASP-2 <13.4 relative units and GUDCA ≤0.07 nmol/mL	11.4 (2.56-51.1)	0.001	11.9 (2.33-60.4)	0.003
Ln MASP-2 ≥13.4 relative units and GUDCA >0.07 nmol/mL	5.82 (0.90-37.7)	0.065	12.2 (1.59-93.1)	0.016
Ln MASP-2 ≥13.4 relative units and GUDCA ≤0.07 nmol/mL	41.4 (8.64-198.5)	<0.001	37.6 (6.92-204.6)	<0.001
RERI	25.2 (-17.4 to 67.7)	0.245	14.6 (-20.2 to 49.3)	0.411
AP	0.61 (0.34 to 0.88)	<0.001	0.39 (-0.16 to 0.94)	0.165
SI	2.65 (1.29 to 5.44)	0.008	1.66 (0.66 to 4.22)	0.285

GDM, gestational diabetes mellitus; MASP, mannan-binding lectin-associated serine protease; DCA, deoxycholic acid; GUDCA, glycoursodeoxycholic acid; OR, odds ratio; CI, confidence interval; RERI, relative excess risk due to interaction; AP, attributable proportion due to interaction; SI, synergy index.

Multivariate analysis 1 was adjusted for GUDCA ≤0.07 nmol/mL in testing the additive interaction between MASP-2 and DCA and adjusted for DCA ≤0.28 nmol/mL in testing the additive interaction between MASP-2 and GUDCA.

Multivariate analysis 2 was further adjusted for traditional risk factors, including pre-pregnancy body mass index, family history of diabetes in first-degree relatives, systolic blood pressure, smoke before or during pregnancy, drink before or during pregnancy, weight gain to the time of glucose challenge test, preexisting diseases (including heart diseases, nephritis, hepatitis, hyperthyroidism, anemia, and tumor), and multiple pregnancies.

### 3.4 Mediation effect of LPC18:0 for the copresence of high MASP-2 and low DCA for GDM

The copresence of high MASP-2 and low DCA was associated with a markedly increased risk of high LPC18:0 (i.e., ≥18.0 nmol/mL) (adjusted OR: 4.28, 95% CI: 2.24–8.18). After adjustment for LPC18:0, the OR of the copresence of high MASP-2 and low DCA for GDM was greatly attenuated from 10.6 (95% CI: 4.22–26.4) to 4.66 (1.41–15.4). The mediation effect of LPC18:0 on the risk association of the copresence of high MASP-2 and low DCA with GDM was statistically significant (*P* for Sobel test <0.001) (**Tables 3**, **4**). High LPC18:0 also played a significant mediating role in the association between low DCA and GDM (*P* for Sobel test <0.001) but not in the association between high MASP-2 and GDM because the adjustment for LPC18:0 increased the OR of high vs. low MASP-2 for GDM from 3.12 to 3.48 (**Tables 2**, **4**).

**Table 4:** Mediation effect of LPC18:0 on risk associations from MASP-2, low DCA and both to GDM.

	Beta (SD)	OR (95% CI)	*P* value
Mediation effect of LPC18:0 for interaction between MASP-2 and DCA			
Model A (LPC18:0 ≥18.0 nmol/mL as the outcome)^†^			
Ln MASP-2 ≥13.4 relative units and DCA ≤0.28 nmol/mL	1.45 (0.33)	4.28 (2.24-8.18)	<0.001
Model B (GDM as the outcome)			
LPC18:0 ≥ vs. <18.0 nmol/mL	2.95 (0.40)	19.1 (8.78-41.7)	<0.001
Model C (GDM as the outcome)^‡^			
Ln MASP-2 ≥13.4 relative units and DCA ≤0.28 nmol/mL	1.54 (0.61)	4.66 (1.41-15.4)	0.012
Sobel test for mediation effect^*^			<0.001
Mediation effect of LPC18:0 for DCA			
Model A (LPC18:0 ≥18.0 nmol/mL as the outcome)^†^			
DCA ≤0.28 nmol/mL	0.86 (0.22)	2.36 (1.54-3.62)	<0.001
Model B (GDM as the outcome)			
LPC18:0 ≥ vs. <18.0 nmol/mL	2.95 (0.40)	19.1 (8.78-41.7)	<0.001
Model C (GDM as the outcome)^‡^			
DCA ≤0.28 nmol/mL	0.17 (0.37)	1.18 (0.58-2.43)	0.647
Sobel test for mediation effect^*^			<0.001
Mediation effect of LPC18:0 for MASP-2			
Model A (LPC18:0 ≥18.0 nmol/mL as the outcome)			
Ln MASP-2 ≥13.4 relative units	0.53 (0.22)	1.70 (1.09-2.64)	0.016
Model B (GDM as the outcome)			
LPC18:0 ≥ vs. <18.0 nmol/mL	2.95 (0.40)	19.1 (8.78-41.7)	<0.001
Model C (GDM as the outcome)^‡^			
Ln MASP-2 ≥13.4 relative units	1.25 (0.43)	3.48 (1.50-8.07)	0.004
Sobel test for mediation effect^*^			0.026

GDM, gestational diabetes mellitus; MASP, mannan-binding lectin-associated serine protease; DCA, deoxycholic acid; LPC, lysophosphatidylcholine; SD, standard definition; OR, odds ratio; CI, confidence interval.

All models were adjusted for traditional risk factors, including pre-pregnancy body mass index, family history of diabetes in first-degree relatives, systolic blood pressure, smoke before or during pregnancy, drink before or during pregnancy, weight gain to the time of glucose challenge test, preexisting diseases (including heart diseases, nephritis, hepatitis, hyperthyroidism, anemia, and tumor), and multiple pregnancies.

^†^Further adjusted for GUDCA ≤0.07 nmol/mL.

^‡^Further adjusted for LPC18:0 ≥18.0 nmol/mL.

^*^ *P* value for Sobel test <0.05 indicating a significant mediation effect.

### 3.5 Predictive values of MASP-1 and MASP-2 in the diagnosis of GDM

Inclusion of MASP-1 and MASP-2 significantly increased the area under the ROC curve (AUC) from 0.68 (95% CI: 0.63–0.73) for the model incorporating traditional risk factors only to 0.71 (0.66–0.76) (*P* < 0.05). Similarly, inclusion of MASP-1 and MASP-2 also increased the AUC of a model including traditional risk factors plus BAs (DCA and GUDCA) from 0.70 (0.65–0.75) to 0.74 (0.69–0.79) (*P* < 0.05) (**Supplementary Figure S1**).

## 4 Conclusion

In this nested case-control study, we found that high MASP-1 and MASP-2 in early pregnancy were associated with an increased risk of GDM in Chinese pregnant women, with the effect of MASP-1 being accounted for by MASP-2. There was a significant interaction between high MASP-2 and low DCA for an increased risk of GDM. The interactive effect between high MASP-2 and low DCA for GDM was partially mediated via high LPC18:0.

Overactivation of the complement lectin pathway causes overproduction of pro-inflammatory cytokines and inflammation, leading to exacerbated tissue injury, insulin resistance, and several chronic inflammatory diseases (24). Some studies have previously explored associations of MASPs with the risks of prediabetes and diabetes, but their findings were inconsistent and inconclusive. A cross-sectional study (n = 439) from Augsburg, Germany, observed that high MASP-1 was independently associated with prediabetes but was not associated with type 2 diabetes (10). While an age- and gender-matched case-control study (n = 200) from Aarhus, Denmark, reported that increased MASP-1 was positively associated with type 2 diabetes (11). However, none of them have assessed the risk associations of MASP-2 with diabetes. To our knowledge, our study was the first to report associations of MASP-1 and MASP-2 in early pregnancy with an increased risk of GDM. In this case-control study, we found that high MASP-2 in early pregnancy was independently associated with a markedly increased risk of GDM, while the effect of high MASP-1 in early pregnancy on GDM was attributable to high MASP-2.

It is biologically plausible that MASP-1 indirectly activates the lectin pathway via promoting MASP-2 activation. A study of a mouse strain lacking MASP-2 found that only MASP-2 can cleave C4 and C4b-bound C2 to form lectin pathway C3 and C5 convertase complexes C4b2a and C4b2a(C3b)n, thus initiating complement activation (25). MASP-1 alone fails to cleave C4 but may facilitate MASP-2 activation through cleaving C2 (26). Hence, lectin pathway activation is deficient in the absence of MASP-2.

GDM is typically characterized by insulin resistance and β-cell dysfunction during pregnancy. Several lines of evidence support that overactivation of the complement lectin pathway in early pregnancy is associated with an elevated risk of GDM through inhibiting β-cell function and exacerbating insulin resistance. One possible mechanism is that complement activation produces several inflammatory mediators, including anaphylatoxins C3a and C5a, and augments inflammasome activation (27). On the one hand, inflammasome activates the production of interleukin-1β (IL-1β), which can directly inhibit islet β-cell function, upregulate islet Fas death receptor, and promote apoptosis (28, 29). On the other hand, macrophages infiltrate adipose tissue and polarizes into M1 cells in response to signaling via C3a-C3aR and C5a-C5aR axes, thereby exacerbating insulin resistance (9). An animal study showed that knockout of C3aR or C5aR in high-fat diet mice reduced macrophage infiltration and improved insulin sensitivity (30, 31). Another possible mechanism is that C3a-desArg/acylation-stimulating protein (ASP), the C3 cleavage product, can stimulate adipocyte glucose uptake and enhance glucose-stimulated insulin secretion through acting directly on islet β cells (32, 33). *In vivo* experiments demonstrated that mice lacking ASP presented decreased adipose tissue and leptin levels and elevated insulin sensitivity (34).

It is quite interesting to note that there was a significant additive interaction between high MASP-2 and low DCA toward increasing the risk of GDM. We also observed that LPC18:0 mediated the interactive effect of high MASP-2 and low DCA on the risk of GDM. First, it is biologically plausible that low DCA plays a critical role in the link between high MASP-2 and GDM. High MASP-2 contributes to hyperactivation of the complement cascade that may promote adipose tissue inflammation and insulin resistance via stimulation of macrophage infiltration and M1 polarization (9). It is also known that DCA plays a critical role in modulating lipid and glucose metabolism, adipose tissue inflammation, and insulin resistance by activating Farnesoid X receptor (FXR) and G-protein-coupled bile acid receptor (TGR5) (35, 36). Hence, it is speculative that high MASP-2 and low DCA have a synergistic effect on the increased risk of GDM through promoting adipose tissue inflammation and insulin signaling dysfunction. Second, available evidence supports that LPC18:0 plays a role in the pathway from additive interaction of high MASP-2 and low DCA to an increased risk of GDM. An animal study found that increased intestinal tauro-β-muricholic acid, an antagonist of FXR, attenuated the LPC increase in the high-fat diet-induced mouse (37). It is possible that low DCA increases LPCs through the FXR signaling pathway. In addition, high LPCs can increase oxidative stress through inducing the overproduction of nitric oxide and release of inflammatory mediators in adipocytes, resulting in increased insulin resistance and GDM (38, 39). In this connection, our mediation analysis found that LPC18:0 played a significant mediating role in the links from DCA to GDM and from the copresence of DCA and MASP-2 to GDM but not in the link from MASP-2 to GDM. Hence, our data support that high MASP-2 amplifies the risk of low DCA for GDM, which is mediated via LPC18:0.

The major strength of our study is that blood samples of pregnant women were collected in early pregnancy (at a median of 10th gestational weeks) much earlier than the time of GDM diagnosis (24th–28th gestational weeks), and thus, a reverse causation was unlikely. Another strength of the study was that traditional risk factors for GDM were carefully collected in our cohort and were adjusted in the current analysis in order to minimize the effect of confounders. Our study also has several limitations. First, only relative concentrations of serum MASPs were available to our analysis, and absolute MASP-2 concentrations need to be measured in future validation studies for possible translation into clinical practice. Second, dietary habits during pregnancy were not collected due to a busy clinical setting. However, dietary intake is more likely to be a cause of serum proteins but less likely to be a consequence of serum proteins. Third, our study women were from urban Tianjin, China, and further studies in other Chinese and non-Chinese populations are warranted to validate our findings. Molecular mechanisms underlying the roles of interplays among MASPs, DCA, and LPC18:0 in the etiology of GDM need further investigations in the future.

In conclusion, we found that high MASPs in early pregnancy were associated with a markedly elevated risk of GDM in Chinese pregnant women, with the effect of MASP-1 on GDM being attributable to serum levels of MASP-2. High MASP-2 and low DCA had a significant additive interaction toward increasing the risk of GDM. The additive interactive effect between high MASP-2 and low DCA on GDM was partially mediated via LPC18:0. The identified biomarkers may have predictive values for GDM in early pregnancy and can be utilized as an early pregnancy predictor for GDM. The additive interaction may be useful for specific interventions if replication studies can confirm our findings in other Chinese and non-Chinese populations. Further mechanistic studies are also needed to elucidate the underlying molecular mechanisms underlying these interesting findings.

## Data availability statement

The datasets used and/or analyzed during the current study are available from the corresponding author on reasonable request. Requests to access these datasets should be directed to yangxilin@tmu.edu.cn or yxl@hotmail.com.

## Ethics statement

The study protocol was approved by the Ethics Committee for Clinical Research of Tianjin Women and Children’s Health Centre (TWCHC) and written informed consent was obtained from participants before data collection.

## Author contributions 

XY, JHL, GH, ZY, and ZF conceived the idea and designed the study; WL, SZ, PW, and JHL collected the data; MG analyzed the data; MG and JL wrote the first draft. All others gave critical comments and edited the manuscript. All authors gave comments and contributed to the writing of the manuscript and agreed to submit and publish the manuscript. XY and MG took full responsibility for the work as a whole, including the study design, access to the data, and decision to submit. All authors contributed to the article and approved the submitted version.
