# Supplementary material for: Serum mannan-binding lectin-associated serine proteases in early pregnancy for gestational diabetes in Chinese pregnant women
Source: Front Endocrinol (Lausanne). 2023 Oct 24;14:1230244. doi: 10.3389/fendo.2023.1230244 (PMC10628726; doi:10.3389/fendo.2023.1230244)
Supplement: Supplementary file 1 [file DataSheet_1.pdf]

Supplementary Table S1 Mediation effect of MASP-2 for MASP-1 to increased risk of GDM.

	Beta (SD)	OR (95% CI)	*P* value
Model A (Ln MASP-2 ≥13.4 relative units as the outcome)			
Ln MASP-1 ≥14.1 relative units	2.19 (0.26)	8.92 (5.33-14.9)	<0.001
Model B (GDM as the outcome)			
Ln MASP-2 ≥13.4 relative units	1.14 (0.27)	3.12 (1.82-5.35)	<0.001
Model C (GDM as the outcome)			
Ln MASP-1 ≥14.1 relative units	0.32 (0.26)	1.37 (0.86-2.29)	0.221
Sobel test for mediation effect*			0.003

Abbreviations: GDM, gestational diabetes mellitus; MASP, mannan-binding lectin-associated serine protease; SD, standard definition; OR, odds ratio; CI, confidence interval.

Model A and Model B were adjusted for traditional risk factors, including pre-pregnancy body mass index, family history of diabetes in first-degree relatives, systolic blood pressure, smoke before or during pregnancy, drink before or during pregnancy, weight gain to the time of glucose challenge test, preexisting diseases (including heart diseases, nephritis, hepatitis, hyperthyroidism, anemia and tumor) and multiple pregnancies.

Model C was further adjusted for Ln MASP-2 ≥13.4 relative units in addition to the variables listed in Model A and Model B.

**P* value for Sobel test <0.05 indicating significant mediation effect.

Supplementary Figure S1 Receiver operating characteristic curves of traditional risk factors, bile acids (BAs) plus mannan-binding lectin-associated serine proteases (MASPs) for gestational diabetes mellitus (GDM) in Chinese pregnant women.

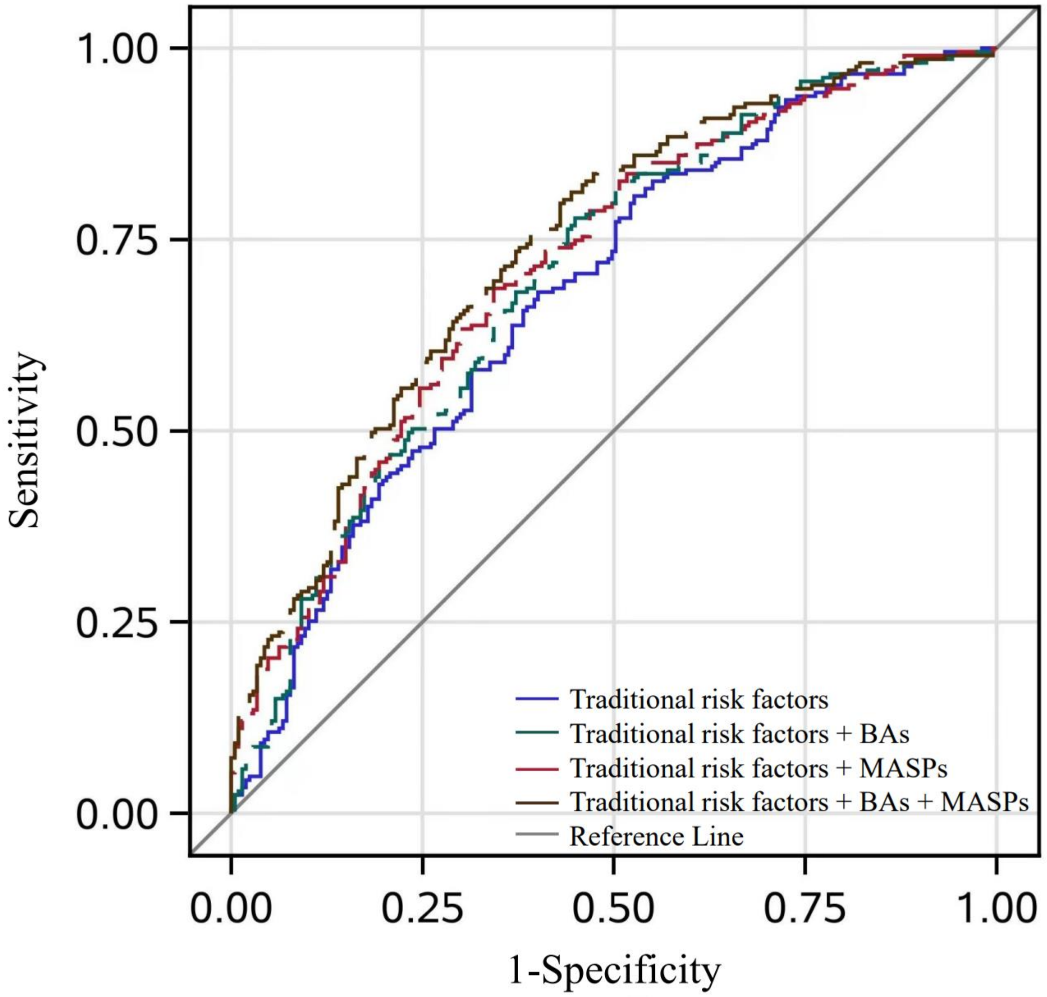

Legends:

MASPs include MASP-1 and MASP-2; BAs include deoxycholic acid (DCA) and glycoursodeoxycholic acid (GUDCA).

The blue (bottom) curve for the traditional risk factors model (model 1), the green (midbottom) curve for the traditional risk factors plus BAs model (model 2), the red (midupper) curve for the traditional risk factors plus MASPs model (model 3), and the brown (upper) curve for the traditional risk factors plus BAs and MASPs model (model 4). The area under curve of those 4 models was 0.68 (95% CI: 0.63-0.73), 0.70 (0.65-0.75), 0.71 (0.66-0.76), and 0.74 (0.69-0.79), respectively. *P* values were less than 0.01 between model 1 and model 4, as well as between model 3 and model 4, and less than 0.05 between model 1 and model 2, between model 1 and model 3, as well as between model 2 and model 4.
